# Recombinant human BMPs 2 and 4 expressed in mammalian cells aiming at bone tissue engineering and stem cell proliferation and differentiation

**DOI:** 10.1186/1753-6561-8-S4-P84

**Published:** 2014-10-01

**Authors:** Ana Claudia O Carreira, Erik Halcsik, Paula Mota de Sá, Felipe Zattar, José Mauro Granjeiro, Mari Cleide Sogayar

**Affiliations:** 1 Chemistry Institute, Biochemistry Department; and Cell and Molecular Therapy Center NUCEL-NETCEM, School of Medicine; University of São Paulo, São Paulo, SP; Brazil; 2 Bioengineering Division, National Institute of Metrology, Quality, and Technology, Duque de Caxias, RJ, Brazil; and Department of Dental Materials, Dental School, Fluminense Federal University, Niteroi, RJ, Brazil

## Background

Bone Morphogenetic Proteins (BMPs) are multifunctional, secreted cytokines belonging to the TGF-β superfamily. These proteins act as a disulfide-linked homodimer, being potent regulators of bone and cartilage formation and repair, cell proliferation in embryonic development and adult bone homeostasis. BMPs are promising molecules in periodontal regeneration to treat physiopathological bone loss and non-union fractures and in oral surgery, and to accelerate and increase osseointegration. BMPs are dimeric molecules displaying sites for N- and O-glycosylation, which increases the stability and half-life of the protein in the body, in addition to determining the specificity of receptor coupling. BMP-2 induces cartilage and bone formation. BMP4 has also been shown to play a role in triggering osteoblastic differentiation of mesenchymal stem cells, through activation of osteoblastic related genes. In order to ensure proper glycosylation and conformational folding and to prevent immunogenicity, we elected a mammalian cell expression system to produce these BMPs aiming at bone regeneration, stem cell proliferation and differentiation and their application in human and veterinary cell therapy.

## Methods

BMPs 2 and 4 cDNAs were amplified from an in-house constructed cDNA Bank and cloned into the pGEM^®^-T-Easy vector. *E. coli *transformants were screened by colony PCR. Upon DNA sequencing, the BMP 2 and 4 inserts were transferred to a lentiviral expression vector. HEK293 cells were co-transfected with a lentiviral plasmid containing both BMP 2 or 4 and eGFP cDNAs, by co-transfection, at a 40:1 ratio, with a Hygro^r ^vector for clone selection. Cell clones were selected using 100 ug/mL hygromicin. Several cell clones were characterized and highest overproducing ones were selected for each protein. BMPs expression was analyzed by qRT-PCR, Western blotting, and *in vitro *biological activity by alkaline phosphatase activity in C2C12 cells during 7 days. Recombinant proteins were purified using heparin affinity chromatography.

## Results and conclusions

Upon cell cloning, most of the cells present in the selected clones were positive for GFP, indicating that a high transfection efficiency was achieved. BMPs 2 and 4 were continuously secreted to the medium even after 120h of serum starvation. Purification of rhBMP2 and 4 from the conditioned medium resulted in more than 90% purity. The rhBMPs 2 and 4 bound to the resin were eluted in 450mM NaCl buffer, with a single dimeric 30-37 kDa band being observed in the eluates. *In vitro *assays showed that the purified rhBMPs 2 and 4 displayed high osteogenic activity. The *in vivo *osteogenic bioactivity analysis of the purified proteins by ectopic bone formation using Rowett rats is underway. Glycosylation analysis using exoglicosidase digestion and structural analysis of the purified proteins is underway. The use of these biopharmaceuticals in bone Tissue Engineering is likely to allow accelerated recovery to both human patients and animals.

## References

[B1] Granjeiro JM Oliveira RC Bustos-Valenzuela JC Sogayar MC Taga R Bone morphogenetic proteins: from structure to clinical use Braz Med Biol Res 2005 38 10 1463 1473 10.1590/s0100-879x2005001000003 16172739

[B2] Bustos-Valenzuela JC Halcsik E Bassi EJ Demasi MA Granjeiro JM Sogayar MC Expression, purification, bioactivity, and partial characterization of a recombinant human bone morphogenetic protein-7 produced in human 293T cells Mol Biotechnol 2010 46 2 118 26 doi: 10.1007/s12033-010-9287-0 20499289 10.1007/s12033-010-9287-0

[B3] Urist MR Nogami H Mikulski A A bone morphogenetic polypeptide Calcified Tissue Research Calcif Tissue Res 1976 21 Suppl 81 7 953844

